# Water and Muscle Contraction

**DOI:** 10.3390/ijms9081435

**Published:** 2008-08-18

**Authors:** Enrico Grazi

**Affiliations:** Department of Biochemistry and Molecular Biology, Ferrara University, Via Borsari 46, 44100 Ferrara, Italy

**Keywords:** Water, muscle contraction, osmotic pressure, chemical potential, stiffness, viscosity, working stroke

## Abstract

The interaction between water and the protein of the contractile machinery as well as the tendency of these proteins to form geometrically ordered structures provide a link between water and muscle contraction. Protein osmotic pressure is strictly related to the chemical potential of the contractile proteins, to the stiffness of muscle structures and to the viscosity of the sliding of the thin over the thick filaments. Muscle power output and the steady rate of contraction are linked by modulating a single parameter, a viscosity coefficient. Muscle operation is characterized by working strokes of much shorter length and much quicker than in the classical model. As a consequence the force delivered and the stiffness attained by attached cross-bridges is much larger than usually believed.

## 1. Introduction

The sliding-filament theory of muscle contraction [1, 2] is almost universally accepted. The relative motion of thick and thin filaments in the sarcomere is generated by myosin heads which undergo an actin-activated ATPase cycle during which they form transient cross-bridges between the filaments [3, 4]. We are not interested, however, in the details of the mechanism but on the gross features of the contractile apparatus: the non ideality [5], the viscosity [6] and the response time of the system.

Most of this review is concerned with the involvement of water in muscle contraction. The involvement is due to the hydrophilic nature of the proteins of the contractile apparatus. The water solutions of these proteins, with the exception of G-actin (Grazi, unpublished results), are highly non-ideal (Appendix A). The consequence of this property is that a small change of the protein concentration induces a large change of the water chemical potential and consequently an opposite change of the protein chemical potential. The chemical potential of the proteins is related to the stiffness of the contractile structure, a property of first importance in the operation of the contractile apparatus. Furthermore the increase of the chemical potential of the proteins is accompanied by the increase of the viscosity of the solution.

A further point is the ability of the hydrated proteins of the contractile apparatus to assemble spontaneously *in vitro* to form ordered structures. Under proper condition G-actin molecules assemble into the double helical polymeric F-actin and myosin molecules assemble into the bipolar myosin filaments (thick filaments). In the absence of ATP, specific surfaces of the actin monomer and of the head of myosin associate to form actin-myosin the heavier component of the contractile apparatus. This association provides a geometric frame that relates protein osmotic pressure (and protein concentration) both to the inter-filament distance and to the elastic force acting on each protein structure. As a consequence, the change of the volume of the system is linked exclusively to the change of the inter-filament distance and this latter determines the change of the angle formed between the attached cross-bridges and the thick and thin filaments. This constitutes, as a whole, a coherent system, which links the protein osmotic pressure to the elastic reaction of the cross-bridge components.

We will:

1. Describe the non-ideal behaviour of the myosin filaments suspensions.
2. Mimic the behaviour of detached and of attached cross-bridges.
3. Reason on the force-length constant.
4. Describe the osmotic properties of myosin subfragnent-1.
5. Describe the effect of protein osmotic pressure on the stiffness of the attached cross-bridges and on the contractile force.
6. Define the power stroke.
7. Introduce viscosity, an inseparable partner of muscle contraction.

## 2. Results

### 2.1. Non-ideal behaviour of the myosin filaments suspensions

**Figure 1.**
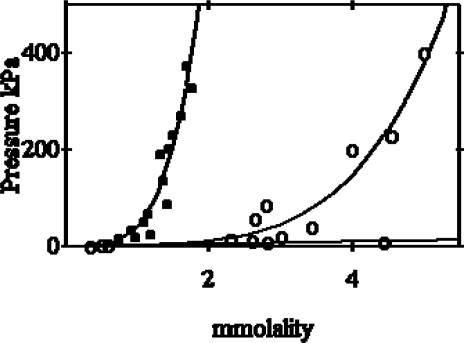
Protein osmotic pressure as a function of the molality of myosin. **(a)** Ideal solution, π = 10^3^ RT *m*, the pressure, in this scale, almost coincides with the bottom line of the figure. **(b)** Myosin, (filled circles): data are fitted by the curve π = 10^3^ RT (*m* + 1.7 × 10^10^*m*^4^). **(c)** Myosin rods, (open circles): data are fitted by the curve π = 10^3^ RT (*m_r_* + 2 × 10^9^ m_r_^4,4^) [7].

The osmotic pressure induced by myosin filament suspensions at various concentrations is illustrated in Figure 1. It is clear the non-ideal behaviour of both myosin (filled circles) and of myosin rods (open circles). In fact the two curves do not obey the expression,


$$\pi \;=10^{3}\;\text{RT}\;\text{m}$$

that relates osmotic pressure, π, and solute concentration, m, in an ideal solution.

**Figure 2.**
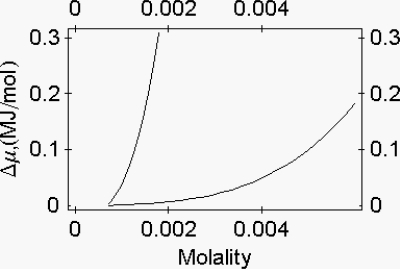
Chemical potential change of myosin and of the myosin rods as a function of their molality. **(a)** The Δμ of myosin (left curve) is calculated by making use of the equation μ_m_ = RT (2.26666 × 10^10^*m*^3^ + ln[*m*]) + cost. **(b)** The Δμ of myosin rods (right curve) is calculated by making use of the equation *m_r_* = RT (2.58823 × 10^9^*m_r_*^3,4^ + ln[*m*r])+costant. The molality of reference is 0.72 × 10^−3^ molal [7].

The relation between protein osmotic pressure and protein molality (Appendix A) allows to calculate the chemical potential change of myosin as a function of myosin molality (Figure 2).

### 2.2. Mimicking the behaviour of the detached and attached cross-bridges

**Figure 3.**
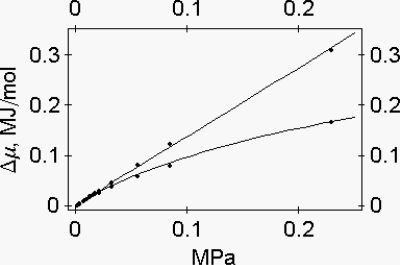
Energy profile of myosin and of the 1:1 actin-myosin complex as a function of protein osmotic pressure. **(a)** Myosin, upper line. Data are fitted by the equation: Δμ = 3.41389 (π – 0.17 × 10^4^)^0.91^ + 2.3988 × 10^−3^ (π – 0.17 × 10^5^)^1.38^ Joule/mol. **(b)** 1:1 actin-myosin complex, lower line. Data are fitted by the equation: Δμ = 4.31613 (π – 0.17 × 10^4^)^0.91^ − 0.0245796 (π − 0.17 × 10^4^)^1.27^ Joule/mol [8].

Suspensions of myosin filaments and of the 1:1 actin-myosin complex were used as a model for detached and for attached cross-bridges, respectively. The two suspensions present distinct energy profiles as a function of protein osmotic pressure (Figure 3).

From these energy profiles we may present the hypothesis that, in muscle: 1). detached cross-bridges change significantly their free energy when sarcomere is shifting from the relaxed to the active or to the rigor state; 2). the cross-bridge attachment-detachment process is accompanied by changes of the muscle protein osmotic pressure [8]; 3). the shift of myosin into the 1:1 actin-myosin complex is controlled by the protein osmotic pressure.

Let us operate at 18 kPa, the putative protein osmotic pressure of *relaxed muscle* [9]. The phenomenological equation that relates protein osmotic pressure to myosin molality is:


(1) $$\pi \;=2\text{.45}\;\times \;\;10^{6}(m_{m}+100\;m_{m}^{2}+1.7\;\;\times \;\;10^{10}m_{m}^{4})\text{Pa}$$

thus the concentration of myosin that generates the pressure of 18 kPa is 7.87 × 10^−4^ molal.

Once actin-myosin is formed the same total myosin molality (in actin-myosin) generates the lower protein osmotic pressure of 12.9 kPa, since the equation that describes the protein osmotic pressure of actin-myosin is:


(2) $$\pi \;=2\text{.45}\;\times \;\;10^{6}(m_{m}+2600\;m_{m}^{2}+7\;\times \;\;10^{9}m_{m}^{3.99}),\text{Pa}$$

If, on the contrary, 18 kPa is the protein osmotic pressure of *muscle in rigor*, the corresponding total myosin molality is 8.79 × 10^−4^ molal (Eq. 2). The same concentration of myosin, after relaxation, Eq. (1) generates the protein osmotic pressure of 27.2 kPa. Thus, actin-myosin formation has a clearly detectable influence on muscle protein osmotic pressure, i.e. on the water chemical potential of the highly non-ideal solution that is the contractile apparatus. Alteration of the water chemical potential necessarily influences the energetics of all the contractile structures, including those complexed with ATP, which cannot be studied by means of systems at the equilibrium.

### 2.3. The force-length constant

**Figure 4.**
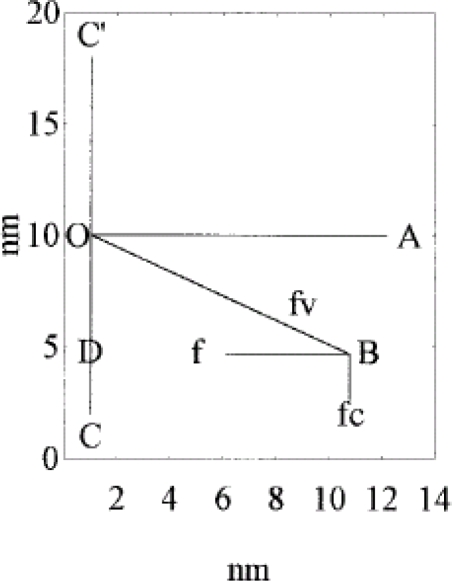
Cross-bridge orientation in one half of the myosin filament. CC’ is the axis of the myosin filament, where C’ is toward the end of the filament and C is toward the middle of the filament. *r*_0_ = OA and AOC = 90° are the radius of the myosin filament and the angle α at protein osmotic pressure ∼0; *r* = BD = BO sin(BOC) and BOC are the radius of the myosin filament and the angle α at the experimental protein osmotic pressure; *F* is the force orthogonal to the filament axis, acting on each cross-bridge; *F_V_* is the component directed toward the constraint; *F*C is the component parallel to the filament axis and directed toward the center of the filament [7].

**Figure 5.**
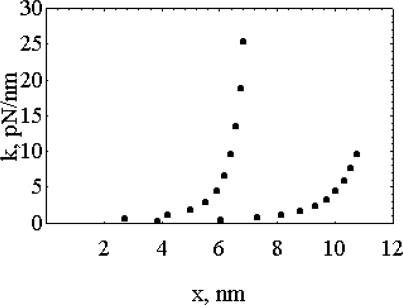
The “force length constant”, *k*=*F_C_/x*, as a function of the distortion. **(a)** First model: lower trace; **(b)** second model, upper trace [7].

The force, *F*, exerted by the cross-bridge along the direction of the filament, is usually considered to be a function only of the position of the base of the cross-bridge relative to the position of its current site of attachment. These relative positions are measured by the variable, *x*, referred to as the “distortion” of the cross-bridge and defined so that *F*(0) = 0. *F*(*x*) is usually taken as a linear function containing a *force-length constant*, *k: F*(*x*) = *kx* [10]. Our studies on the osmotic properties of myosin filaments suspensions [7] indicate that force, *F*, is not at all a linear function of the deformation, *x*, and that the “length-force constant” changes significantly with the deformation as well as with the length of the rotating arm. In fact by relating the osmotic behaviour (water chemical potential changes), the elastic behaviour (protein chemical potential changes) and the external work applied to the cross-bridges of the myosin filament suspensions it is recognized that:

1. Beyond a given myosin concentration (or a given protein osmotic pressure), any change of the volume of the solution is accompanied by a change of the volume of the hydrated filament, thus of the radius of the hydrated filament. This sets a relationship between the molality of myosin and the radius of the hydrated filament.
2. An equivalence can be set between pressure-volume work and elastic stress and, on the assumption that stress is mostly localized to the cross-bridges, the dependence of cross-bridge distortion on myosin concentration (or protein osmotic pressure) can be calculated.
3. It is found that, *k*, the “force length constant” increases significantly with cross-bridge distortion, *x*, and that the increase depends on the length of the rotating arm. Two models of the rotating arm are selected (Figure 4). The first model assumes that the rotating arm equals *r*_0_. In this case *r* = *r*0 × sin(a). The second model assumes that the length of the rotating arm is half of the difference between the cross-bridge diameter (30.1 nm) and the shaft diameter (15 nm), thus equals 7.55 nm. In this case sin(a) = (*r − b*)/(*r*_0_*− b*), where *b = r*_0_ − 7.55 nm. By increasing protein osmotic pressure from 18 kPa to 50 kPa, the likely range of protein osmotic pressure in muscle, *k*, increases from 0.5 to 1.39 pN/nm for model I (rotating arm 15.05 nm) and from 0.79 to 3.48 pN/nm for model II (rotating arm 7.5 nm) (Figure 5).
4. At constant protein osmotic pressure, if an external, non-osmotic force, parallel to the filament axis, is applied to cross-bridges, these structures are deformed and the water activity coefficient is altered. As a consequence, in muscle, passive and active shortening of the sarcomere is expected to promote the change of the water-water and of the water-protein interactions. We thus depict muscle contraction as a chemo-osmo-elastic transduction, where the analysis of the energy partition during the power stroke requires consideration of the osmotic factor in addition to the chemo-elastic ones [7].

### 2.4. The osmotic properties of myosin subfragment-1

From the work of Rayment et al. [11] myosin subfragment-1 appears as a highly asymmetric particle of 18.5 nm length, 6.5 nm width and approximately 4 nm thickness. The shape of a protein, however is likely to change with a change in the water chemical potential due to a change of protein concentration (protein osmotic pressure). To detect eventual changes, since in concentrated solutions of myosin subfragment-1a regular packing is not easily discernible, we used the scaled particle theory of fluid mixtures [12, 13]. Four subfragment-1 models are considered: (a) a cylinder of 16 nm length and 1.66 nm radius (fully extended conformation). (b) a cylinder of 8 nm length a 2.22 nm radius (partially bent conformation), (c) a spheroid of 3.21 nm radius (completely bent conformation), and (d) a dimeric spheroid of 4.047 nm radius. The activity coefficient of either the monomeric cylinder or the monomeric or dimeric spheroid is calculated according to the scaled particle of fluid mixtures as indicated by Minton [14]. The activity coefficients, which are obtained in the molar (M) scale, are converted into the molal (*m*) scale as indicated by Glasstone [15].

The experiments were performed either in 100 m*m* KCl solutions or in 25 m*m* orthophosphate solutions plus 2 m*m* MgADP. This last condition was used in the attempt to accumulate the subfragment-1 – MgADP – Pi intermediate [4].

**Figure 6.**
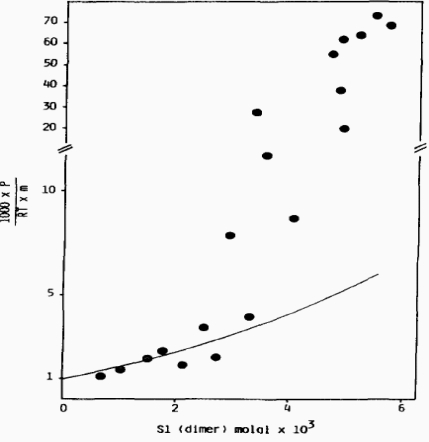
Plot of (1000 π) / (RT *m*) against the molality of subfragment-1 (as dimer) in KCl solutions. Theoretic behaviour calculated according to the scaled particle theory on the assumption that subfragment-1 is a dimer of spheroidal shape and radius 4.047 nm (___) [18].

In KCl solutions (KCl, 0.1 mol; triethanolamine, 0.01 mol; MgCl_2_, NaN_3_ and 2-mercaptoethanol, 2 mmol each, pH, 7.45), between 0.6 and 2.5 m*m*, subfragment-1, behaves like a dimeric spheroid of 4.05 nm radius and a dimerization constant higher than 3.5 × 10^4^*m*^−1^ (Figure 6). This indicates that dimerization occurs with bending of the molecule. Dimerization of subfragment-1 was previously reported to occur at low temperature and at low protein concentration [16]. The identity of the dimeric form and of the refractory states of subfragment-1 was also proposed [17]. Since, in muscle, myosin heads are constrained in their orientation and aggregation state by the myofilament lattice, it is uncertain whether dimers will form *in vivo*. Bending of the head, however, is expected to occur also *in vivo*.

**Figure 7.**
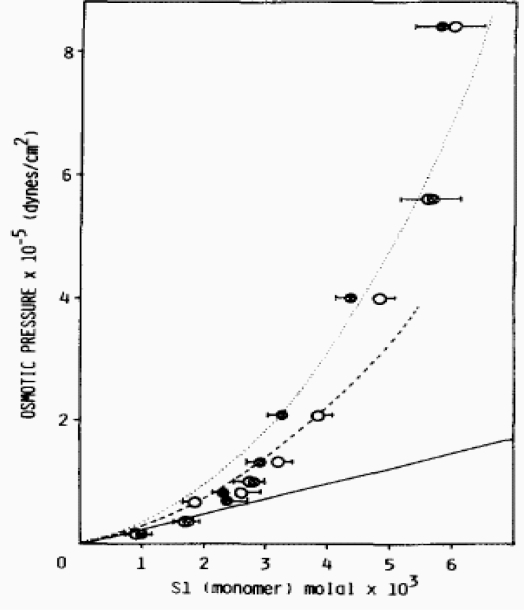
Plot of the protein osmotic pressure of subfragment-1 solutions in orthophosphate, with (open circles) and without MgADP (filled circles). **(a),** Ideal behaviour, 1000 π / RT = *m,* (___); **(b),** theoretical behaviour calculated on the assumption that subfragment-1 is a cylinder of 9 nm length and 2.22 nm radius (.....); **(c)**, and on the assumption that subfragment-1 is a sphere of 3.21 nm radius (-----) [18].

In 25 m*m* orthophospate solutions, up to the protein osmotic pressure of 10 kPa, the subfragment-1 particle displays essentially the same behaviour in both the presence and in the absence of MgADP. In contrast, between 10 and 40 kPa, the behaviour differs significantly. In particular at 18 kPa, the protein osmotic pressure in frog muscle [9], in the absence of MgADP, subfragment-1 behaves like a monomeric cylinder with a height to diameter ratio of 2.07, while in the presence of MgADP, it behaves like a monomeric spheroid. According to the scaled particle theory, our results indicate that in muscle the myosin head is not fully extended. It is bent and bending is increased in the myosin-MgADP-Pi intermediate (Figure 7) [18].

### 2.5. Protein osmotic pressure, stiffness of the attached cross-bridges and contractile force

Osmotic pressure, π, is the rate of change of energy in relation to the volume of all the exchangeable species. Thus changing the volume fraction or the concentration of the macromolecular species by applying osmotic pressure is physical work done on that species. This work can be expressed as the chemical potential of the macromolecular subject to stress at fixed temperature, T, hydrostatic pressure, p, and activities, n_i_, of small molecules:


$$\Delta \mu (\text{T},\text{p},{\text{n}}_{\text{i}})\;=-\pi \;\Delta \text{V}\;\;(\text{Joule})$$

where, V is the total volume, essentially the water volume that moves to or from the phase of interest [19]. Thus when a protein osmotic stress is applied to F-actin or to subfragment-1 decorated F-actin osmotic work is transformed into mechanical work that compresses the filament. This occurs because, concomitantly with the decrease of the volume of the solution, the macromolecular protein osmotic pressure and the distortion of the contractile structures increase, thus changes the orientation of the actin monomer in F-actin, in tropomyosin-F-actin, in the myosin subfragment-I decorated F-actin and in the myosin subfragment-1 decorated tropomyosin-F-actin (Appendix B and C).

The distortion of the contractile structures increases their stiffness and, therefore, their elastic moduli by bending. In particular, at the protein osmotic pressure of 18 kPa, the monomer in F-actin and in tropomyosin-F-actin display an elastic modulus by bending of 4.74 MPa and of 5.8 MPa, respectively.

Decoration of these structures with myosin subfragment-1 increases significantly the elastic modulus by bending of the monomer. At the protein osmotic pressure of 18 kPa the elastic modulus by bending for myosin subfragment-1 decorated F-actin is 22MPa and for myosin subfragment-1 decorated tropomyosin-F-actin is 22.3MPa.

The increase from 4.74 to 22 MPa of the rigidity of the monomer of F-actin, following the decoration with myosin subfragment-1, is compatible with the development of a force of 3.96 pN per monomer, a force correctly oriented to promote the sliding of the actin filament toward the center of the sarcomere. The magnitude of this force is comparable to the average force developed by a single cross-bridge in intact skeletal muscle [20, 21]. In contrast, the myosin subfragment-1 decorated tropomyosin-F-actin rigor complex develops a much smaller driving force that favours relaxation. Apparently tropomyosin uncouples the osmotic and the mechanical event.

We thus propose that the energy for muscle contraction is stored as elastic energy in the actin filament and in the cross-bridge. The elastic energy is provided by protein osmotic pressure. We have shown that the stiffness of the cross-bridge components increases with protein osmotic pressure. Since the contractile force cannot be larger than the stiffness of the cross-bridge, it follows that the magnitude of the contractile force increases with protein osmotic pressure [22, 23].

### 2.6. The working stroke

Originally the cross-bridge step size was fixed at 15 nm [24]. Worthington and Elliott with their impulsive force theory [25, 26] criticized this choice. They proposed that cross-bridge step size is smaller, 2 nm, and of variable length. This view found experimental support from the work of Reconditi *et al.* [27] who showed that working stroke is smaller and slower at higher load. In these experiments a load step of 150 μs is first applied to the fibre (phase 1) and is accompanied by fibre shortening because of the compliance of the myosin heads and the actin and myosin filaments. After the load step rapid shortening continues for a few milliseconds. This phase 2 shortening is thought to be due to the working stroke. A filament sliding of 5.2, 6.4 and 8.1 nm per half sarcomere occurs after load steps to 0.75, 0.50 and 0.25T_0_, respectively.

**Figure 8.**
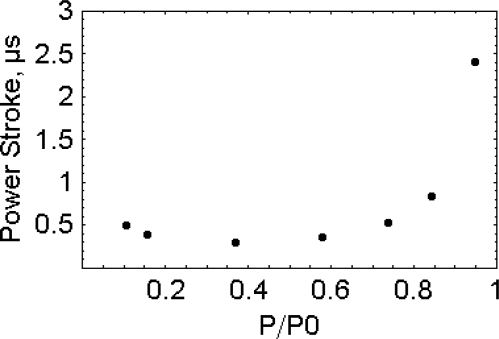
Estimated time length of the working stroke as a function of the load at steady fibre shortening. Elaborated, according to [6], from the data of [28].

**Figure 9.**
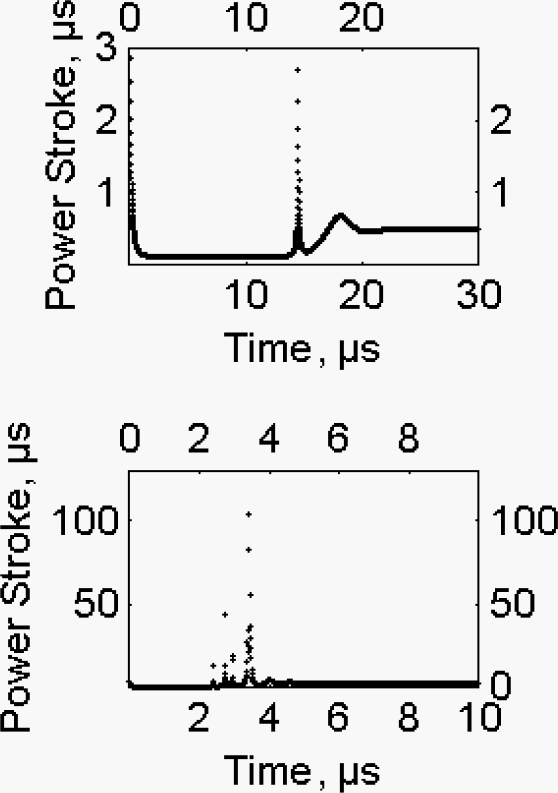
Pre-steady fibre shortening, estimated time length of the working stroke. Elaborated, according to [6], from the data of He et al. [28]. **(a)** Upper figure, 0.105 P/P_0_; **(b)** Lower figure, 0.947 P/P_0_.

In our model [6] the power output is defined as the ATPase rate [28] time the number of the myosin head per the half sarcomere. Since the working strokes occur randomly [29, 30] they mostly occur one at the time. Thus, in order to contribute to contraction, they must deliver a force at least equal to the contractile force, F_1_, experienced by the half sarcomere at that moment. The energy available to each working stroke, 7.44 × 10^−8^ pJ per molecule (E_ATP_) [31], is the free energy of hydrolysis of ATP in muscle conditions. The maximum length, *l*_M_, possibly spanned in the course of the working stroke is thus, *l*_M_ = E_ATP_/F_1_, and the estimated time length of the working stroke is,

$${\text{t}}_{\text{W}}={\text{E}}_{\text{ATP}}/{\text{F}}_{1}/{\text{V}}_{\text{V}}$$

where v_v_ is the actual shortening rate experienced by the half sarcomere at that moment. During the steady shortening of the fiber the time length of the working stroke is 2.4 μs, 0.296 μs and 0.488 μs at 0.947, 0.368 and 0.105 P/P_0_, respectively, where P, is the tension and P_0_, is the isometric tension (Figure 8). Longer time lengths of the working stroke are experienced in the pre-steady state. At 0.105 P/P_0_, (Figure 9, upper part) the time length of the working stroke is 3 μs as compared to 0.488 μs of the steady state. At 0.947 P/P_0_ (Figure 9, lower part) the time length of the working stroke is 100 μs as compared to 2.4 μs of the steady state. Please notice that the pre-steady lasts more than 20 μs at 0.105 P/P_0_ and about 5 μs at 0.947 P/P_0_.

According to He *et al*. [28], at 0.105 and 0.947 P/P_0_, the rates of actin-myosin ATPase are 16.836 s^−1^ and 5.786 s^−1^ and the periods 0.0594 s and 0.173 s, respectively. Thus, according to our model, the time length of the working stroke is only a very minor part of the ATPase period. This means that the available chemical energy is converted very rapidly into mechanical energy and that the force delivered and the stiffness acquired are orders of magnitude larger than those of the classical models. A further consequence is that the stiffness fades with the working stroke therefore the cross-bridges, still attached, oppose very little to the sliding of the filaments. In the pre-steady state the fraction of the attached cross-bridges seems to be larger than in the steady state as judged from the significantly longer time length of the working stroke.

In conclusion our view on the working stroke is definitely different from the classical view, this latter is based on the Huxley-Simmons manoeuvre and on the alleged cross-bridge synchronization, where the working stroke spans a distance of a few nanometers with time lengths of a few milliseconds.

### 2.7. Viscosity, an inseparable partner of muscle contraction

The question whether viscosity is an important component of muscle contraction is debated since many decades. It is clear that in contracting muscle some work is dissipated to overcome a viscous resistance [32–34]. Wide disagreement occurs however on the impact of viscous hindrance. At the extremes, Ernst [35] calculates that the force of the sliding friction is likely to be larger than the isometric force while Huxley [36] proposes that the viscous drag force is only 10^−4^ of the isometric force. In fact activated fibers display a significant internal viscosity that could arise from cross-bridge interaction [37, 38]. Furthermore Elliott and Worthington [39] calculate a hydrodynamic viscous drag of 6 × 10^−5^ kg s^−1^ for an actin filament of frog muscle during contraction.

In our opinion the contribution of viscosity cannot be neglected in the economy of muscle contraction [6].

The energy delivered by each single power stroke induces the displacement of the masses of the half sarcomere (m_1_) and of the associated load (m_2_). Contraction takes place only when the power strokes reach the right frequency, so that not all the energy provided by a power stroke is used up before the following power stroke, performed by another attached cross-bridge, occurs. When this condition is satisfied the half sarcomere shortens by a uniformly accelerated motion.

The uniformly accelerated motion is not the usual motion of the contraction. To convert the uniformly accelerated motion into the observed uniform motion [28] a viscous hindrance is introduced [40, 41]. Thus a hyperbolic form,


$${\text{V}}_{\text{V}}=\text{k}\;{\text{a}}_{\text{d}}\;\text{t}/(\text{k}+\text{t})$$

is assigned to the velocity, v_V_, of the masses, m_1_ and m_2_, which move under the effect of the driving acceleration,


$${\text{a}}_{\text{d}}=({\text{F}}_{1}+{\text{F}}_{2})/({\text{a}}_{1}+{\text{a}}_{2})$$

The reciprocal of the constant, k, defines the viscous hindrance; a_1_ is the acceleration associated to the contractile force, F_1_; and a_2_ is the acceleration associated to, F_2_, the force of the load.

The frequency of the power strokes is given by the number of the molecules of ATP hydrolyzed per second in the half sarcomere [28]. The value of, k, is adjusted to match the experimental velocity [28]

The system is solved numerically. At each cycle the values of, v_V_, a_d_, a_1_, F_1_, change. While approaching the stationary state, a_d_, tends to zero and, v_V_, tend to the exprimental value. The approach to the steady state requires from few microseconds to almost a millisecond depending on the load.

**Figure 10.**
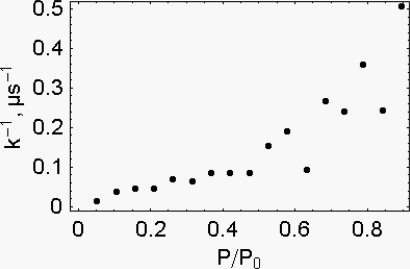
1/k, μs^−1^, as a function of P/P0. Temperature 12 °C. Sarcomere length 2.7 μm [23].

In Figure 10, 1/k, μs^−1^, is shown to increase as a function of P/P_0_. The increase of the load is associated both with the increase of the fraction of the attached cross-bridges and with the decrease of the distance between the sliding surface of the thin and of the thick filaments. It is therefore reasonable that the system experiences an increase of the viscosity coefficient.

According to our model active muscle shortening attains the steady state in a few microseconds, but at very high load the time required is almost a millisecond. This means that, in most cases, the present time resolution is not adequate to observe the pre-steady state, the only state where it is meaningful to investigate the viscous properties of muscle fibers.

## 3. Conclusions

### 3.1. The non ideality of the contractile system

Our experiments were performed *in vitro*, at the equilibrium, with the aim to study the macromolecular osmotic pressure generated by the contractile proteins and to mimic the behaviour of the sarcomere. Under these conditions the change of the water chemical potential (change of osmotic pressure) and of the protein chemical potential could be determined without any assumption and their relation with the stiffness of the protein structures and with the capability to support muscle contraction could be established. In general the solutions of the contractile proteins behaved non ideally, therefore small changes of the volume of the system were accompanied by large changes of the macromolecular osmotic pressure and of the stiffness of the structures. At constant volume the interaction of the contractile proteins also perturbed the macromolecular osmotic properties of the system and the chemical potential of the proteins. At constant pH and ionic strength the change of the concentration of small solutes (change of the micromolecular osmotic pressure) did not significantly alter the macromolecular osmotic pressure unless one of the solutes did not specifically bind to one of the proteins of the system. In this case a “protein cross-talking through osmotic pressure” occurred [44].

Recently muscle contraction and cell volume changes were studied in skeletal muscle with a time resolution of 400/s [45]. It could be helpful to extend these also to the changes of the volume of the sarcomere.

It was proposed that osmotic mechanisms could contribute to the power stroke of myosin. In particular it was pointed out that the pressure of a single molecule (e.g. a phosphate ion) expanding a trap could supply part of the energy required to perform the power stroke [46]. Although plausible these mechanisms are highly hypothetical and bears no relationship with our studies on the macromolecular osmotic pressure of the contractile proteins. In fact our results were obtained in the absence of any assumption.

### 3.2. The viscous properties of the system

The question whether the contractile apparatus is a highly viscous system is debated since a longtime. The problem is to explain how a series of impulsive forces, generated by the splitting of ATP, is converted into the smooth and steady movement of the contraction. In our model [6] the conversion is operated by a viscous hindrance that depends on the condition of the contraction, mostly on the load. The reason why the viscous hindrance is usually overlooked is that it can be detected only in the pre-steady state of the contraction. Unfortunately in most cases the pre-steady state is too fast (a few microseconds) to be detected.

### 3.3. The response time of the system

The present picture of muscle contraction is largely influenced by the time resolution of the equipment available. The transition from a resting to an active fiber is usually not explored. The length (or load) steps applied to the active fiber have, at best, a time length of ∼100 μs. The response of the fiber last more than 1 ms, in this time many hundreds of power strokes take place in the sarcomere. It is thus desperately impossible to dissect the contribution of a single power stroke. It is our opinion that a meaningful representation of the power stroke requires the study of the transition of the muscle fiber from the resting to the active state with a time resolution of the order of the microsecond.

## Appendix A

### Ideal and non ideal solutions

In an ideal solution the relation between osmotic pressure, π, and the water molar fraction, X_W_, is given by:

$$\pi \;=-\text{RT}\;\text{ln}[{\text{X}}_{\text{W}}](\text{Pa})$$

or, in the molal scale:

(A1)
$$\pi \;∼{\text{m}}_{\text{P}}\;\text{RT}({\text{M}}_{\text{W}}/\;{\text{V}}_{\text{W}})\;=\;1000\;{\text{m}}_{\text{P}}\;\text{RT}\;\text{(Pa)}$$

where M_W_ = 0.018 kg is the water molal mass; V_W_ = 18 × 10^−6^ m^3^ is the water partial molal volume. Are called non-ideal the solutions that diverge from this behaviour.

### The chemical potential of a protein as a function of the osmotic pressure

In binary systems composed of water and protein the chemical potential of the protein is calculated by the the Gibbs-Duhem relation:

(A2)
$$n_{w}d\mu_{w}+{\text{n}}_{\text{P}}\;d\mu_{\text{P}}=0$$

where *dμ*_P_ and *dμ*_W_ are the chemical potential changes of protein and water, and *n**_P_* and *n*_W_ are the number of moles of protein and water, respectively; the change of the chemical potential of protein as a function of protein molality, *m*, is:

(A3)
$$\begin{array}{l} d\mu_{\text{P}}\;=-n_{W}/{\text{n}}_{\text{P}}\;d\mu_{\text{W}}\\ d\mu_{\text{P}}\;=\;-55\text{.5555}/m\;d\mu_{\text{W}}\\ \mu_{\text{W}}=\mu^{\text{o}}\text{w}\;+\;\text{RT}\;\text{ln}[{\text{a}}_{\text{W}}]\\ \pi \;\text{x}\;V_{W}=-\text{RT}\;\text{x}\;\text{ln}[{\text{a}}_{\text{W}}]\\ \;\;\;\;\;\;\;\;d\mu_{\text{W}}=-V_{W}\;d\;\pi \end{array}$$

since

$$\mu_{\text{W}}=\mu^{\text{o}}\text{w}\;+\;\text{RT}\;\text{ln}[{\text{a}}_{\text{W}}]$$

and

$$\begin{array}{l} \pi \;\text{x}\;V_{W}=-\text{RT}\;\text{x}\;\text{ln}[{\text{a}}_{\text{W}}]\\ \;\;\;\;\;\;\;\;d\mu_{\text{W}}=-V_{W}d\pi \end{array}$$

and Eq. (A3) becomes

(A4)
$$d\mu_{\text{P}}=55.5555\;/\text{m}\;{\text{V}}_{\text{W}}\;d\;\pi$$

where, *V**_W_* = 18 × 10^−6^ m^3^, is the water partial molar volume, π is the protein osmotic pressure and *a*_W_ is the activity of water.

If the phenomenological expression for, π, as a function of the molality of the protein, is known, equation (A4) can be integrated and the Δμ of the protein, as a function of the molality, is obtained.

## Appendix B

### Calculation of the interfilament distance of the actin filament and of the S1-decorated actin filament

Equilibration of either F-actin or S1-decorated F-actin solutions against poly(ethyleneglycol) solutions of known osmotic pressure withdraws water from the protein compartment until the same macromolecular osmotic pressure is reached in both compartments. Above the macromolecular pressure of ∼8 kPa F-actin and S1-decorated F-actin undergo a filament-to-bundle transition and the majority of the filaments is regularly packed [42]. In the course of the process, water is withdrawn both from the inter-filament and from the intra-filament spaces, so that not only the inter-filament distance decreases (lattice compression in muscle fibres), but also the volume and the diameter of the hydrated filament decrease.

The interfilament distance of the actin filament and of the S1-decorated actin filament is calculated from the molal concentration of actin according to the equation:

(B1)
d={[(ν × b m)+ 1.005 × 10−3]  × 4 / (b × N × 2.73 × 10−9 × 23) }12

where 1.005 × 10^−3^ m^3^ is the water volume plus the salt volume, *ν* is the partial specific volume of the protein, *m* in kDa is the molecular mass, *b* is the number of moles of F-actin (as monomer)/kg water. N is the Avogadro number, and 2.73 × 10^−9^ is the number of meters of filament/actin monomer [43]. Eqn. (B1) vanishes below the protein osmotic pressure of 10^4^ Pa. Below this pressure actin filaments are not homogeneously organized into bundles.

## Appendix C

### The compression of the actin filament subjected to an osmotic stress

In the compression of the actin filament by osmotic stress the free-energy change of the solution in the protein compartment accompanying the simultaneous compression and water movement is

$$dG=nFdR-\Delta PdV_{W}$$

where *n* = *b* × N is the total number of molecules of actin in the filaments; *F* is the force orthogonal to the filament axis, acting on each actin monomer; *R* is the radius of the filament; Δ*P* is the chemical potential difference of water in pressure units; and *V**_W_*, is the water volume. At equilibrium

$$nFdR=\Delta PdV_{W}$$

i.e.

$$F=(\Delta P/n)\;\;\times \;dV_{W}/dR$$

Since the volume of the solution is

(C1)
$$2\sqrt{3}\;R^{2}\;\times \;2.73\;\times \;10^{-9}\;\times \;b\;\times \;\text{N}$$

[42] and the volume of the protein is *ν × m × b,* the water volume is

$$V_{W}=(2\sqrt{3}\;R^{2}\times \;\;2.73\;\;\times \;\;10^{-9}\;\;\times \;\;b\;\;\times \;\;\text{N})-(v\;\;\;\times \;\;\;m\;\;\;\times \;\;\;b)$$

and

$$dV_{W}/dR\;=\;4\sqrt{3}\;\times \;2.73\;\;\times \;10^{-9}\;\times \text{N}\;\times \;b\;\times \;R$$

Thus the force F acting on each actin monomer is

(C2)
$$F=4\;\sqrt{3}\;\times \;2.73\;\times \;10^{-9}\;\times \;R\;\times \;\Delta P(\text{newtons})$$

The force *F* is split into two components: *F**_V_*, directed toward the constraint, and *F**_T_*, parallel to the filament axis and directed toward its pointed end (Figure 4).

(C3)
$$F_{T}=F\;\times \;\text{tan}(90-\alpha )\;(\text{newtons})$$

α, is the angle formed between, *l,* the axis of the monomer and the pointed end of the filament axis and is defined by *R*/*l* = Sin[α].

The force *F**_T_* is balanced by the reaction of the monomer or of the decorated monomer.
